# Breaking professional silos to combat brucellosis: a pilot feasibility and proof-of-concept study of interprofessional education to strengthen healthcare capacity in Côte d'Ivoire

**DOI:** 10.3389/fvets.2026.1742755

**Published:** 2026-07-30

**Authors:** Richard B. Yapi, Clarisse A. Houngbedji, Coletha Mathew, Valentin B. Koné, AbdulHamid S. Lukambagire, Mahama Touré, Gilbert Fokou

**Affiliations:** 1 Centre d'Entomologie Médicale et Vétérinaire, Université Alassane Ouattara, Bouaké, Côte d'Ivoire; 2 Centre Suisse de Recherches Scientifiques en Côte d'Ivoire, Département de Recherche et Développement, Abidjan, Côte d'Ivoire; 3 College of Veterinary Medicine and Biomedical Science, Sokoine University of Agriculture, Morogoro, Tanzania; 4 Institut National de Santé, Publique, Abidjan, Côte d'Ivoire; 5 Kilimanjaro Clinical Research Institute, Moshi, Tanzania

**Keywords:** brucellosis, Côte d'Ivoire, interprofessional education, One Health, pilot study

## Abstract

**Introduction:**

Brucellosis is a neglected zoonotic disease of major public health importance in Côte d'Ivoire, where it remains underrecognized in the human health sector despite prioritization under the Global Health Security Agenda. This study aimed to identify knowledge gaps among health professionals and explore the feasibility of a pilot interprofessional education (IPE) intervention to strengthen One Health collaboration for improved disease recognition and management.

**Methodology:**

The study comprised two phases. First, a baseline cross-sectional survey was conducted in March 2024 among 54 human and animal health professionals in the Hambol region to assess knowledge of brucellosis and other febrile illnesses. Findings informed the development of a one-day IPE training workshop. In the second phase, the intervention was implemented in February 2025 with 22 participants from medical, veterinary, and public health institutions as a pilot feasibility and proof-of-concept exercise. Knowledge was measured before and immediately after training across domains including general understanding, transmission, symptoms, diagnosis, and control to assess short-term learning gain.

**Results:**

The baseline survey showed that 90.7% of respondents were aware of zoonoses, but only 38.8% identified brucellosis, with significantly greater awareness among veterinarians (81.3%) than human healthcare workers (23.3%; p < 0.001). Brucellosis was not cited as a cause of fever or miscarriage. Following the training, significant improvements were in overall knowledge scores (34.5 to 37.0; p < 0.005) and symptom recognition (4.9 to 5.9; p < 0.005), while knowledge related to diagnostic and control remained unchanged.

**Conclusion:**

The findings revealed importance awareness gaps among human healthcare workers and suggested that IPE is a feasible approach for strengthen One Health collaboration and improving knowledge of zoonotic disease. Larger studies with longer-up are needed to assess sustained effects on clinical practice, surveillance, and disease control in Côte d'Ivoire.

## Introduction

Brucellosis is a zoonotic infectious disease of public health concern worldwide (1). In Côte d'Ivoire, the disease has been identified as a priority zoonotic disease to be controlled and eliminated under the Global Health Security Agenda framework. However, a lack of knowledge among human health profession hampers its control. In 2017, the seroprevalence of brucellosis among people at risk of infection in the northern part of the country was 5.3% (2). In 2018, a pilot study conducted in northern Côte d'Ivoire showed the disease is unknown to human healthcare workers. In general, veterinarians have the greatest awareness and knowledge of zoonotic diseases (3, 4). This gap needs to be overcome for full control of the disease.

From our knowledge, no program has been put in place so far to address these gaps in the human health sector, to improve health outcomes of infected people and to inform policies regarding the disease. The transmission of this bacterial infection is mostly rampant in developing countries, where it impairs the lives of human and animals whilst being largely under control or eliminated in western countries (1). Untreated, brucellosis evolves to a chronic form leading to significant disability. The disability burden due to *Brucella* infection in humans is estimated at 0.150 for chronic and localized brucellosis and at 0.190 for acute brucellosis, which make it of equal significance as that of malaria, estimated at 0.135 and AIDS estimated at 0.191 (5).

Humans mainly contract brucellosis through close contact with infected animals and via consumption of unpasteurized dairy products (6). Additionally, human-to-human transmission is possible by breast-feeding and sexual intercourse (7). *Brucella* infection causes febrile illness and in pregnant women, it can result in abortion, stillbirth and premature birth (8). As an occupational illness, pastoral communities, small-scale livestock keepers, animal health workers and slaughterhouse personnel bare the greatest burden of the disease. A major challenge of brucellosis control is its diagnosis (9, 10). In malaria-endemic countries, brucellosis is often confused with malaria or typhoid fever, since these acute febrile illnesses all present with nonspecific flu-like symptoms such as fever, headache, and malaise. Consequently, the disease is under-reported and therefore, only incomplete data are available to policy makers and health advocates. In most primary health care facilities in sub-Saharan Africa, diagnosis and treatment are based on clinical manifestations of diseases, resulting in frequent sub-optimal or inappropriate treatment.

A study published in 2022 in pastoralist settings in Tanzania, has shown that the poor knowledge of human brucellosis amongst frontline human healthcare workers and their ability to diagnose and manage the disease needed to be strengthened for effective surveillance and control of the disease (11). Interprofessional education (IPE) in health has been reckoned as paramount to breaking such a barrier. IPE occurs when two or more professions learn with, and from each other to improve collaboration and the quality of care. As a zoonotic disease, the intervention should be tailored in a holistic approach such as the One Health concept. This concept is a collaborative, multisectoral, and transdisciplinary approach with the goal of achieving health outcomes at the intersection between humans, animals, and plants in a shared environment.

The aim of this work was to break the silos between veterinarians and human healthcare workers to improve the detection and management of human brucellosis. Specifically, the work aimed to (i) identify knowledge gaps among human and animal health professionals regarding brucellosis and (ii) the feasibility and immediate learning outcomes of an interprofessional education intervention (IPE) designed to promote One Health collaboration.

## Method

### Study design, setting, population and sampling

This was a pilot feasibility and proof-of-concept study conducted in two sequential phases: (i) a baseline cross-sectional survey to identify knowledge gaps related to brucellosis among human and animal healthcare workers, and (ii) a pre-post assessment of an interprofessional education (IPE) intervention. The study was designed to explore the feasibility of implementing an IPE approach within a One Health framework and to document immediate changes in participant knowledge following training, rather than to evaluate intervention effectiveness.

**Figure 1:**
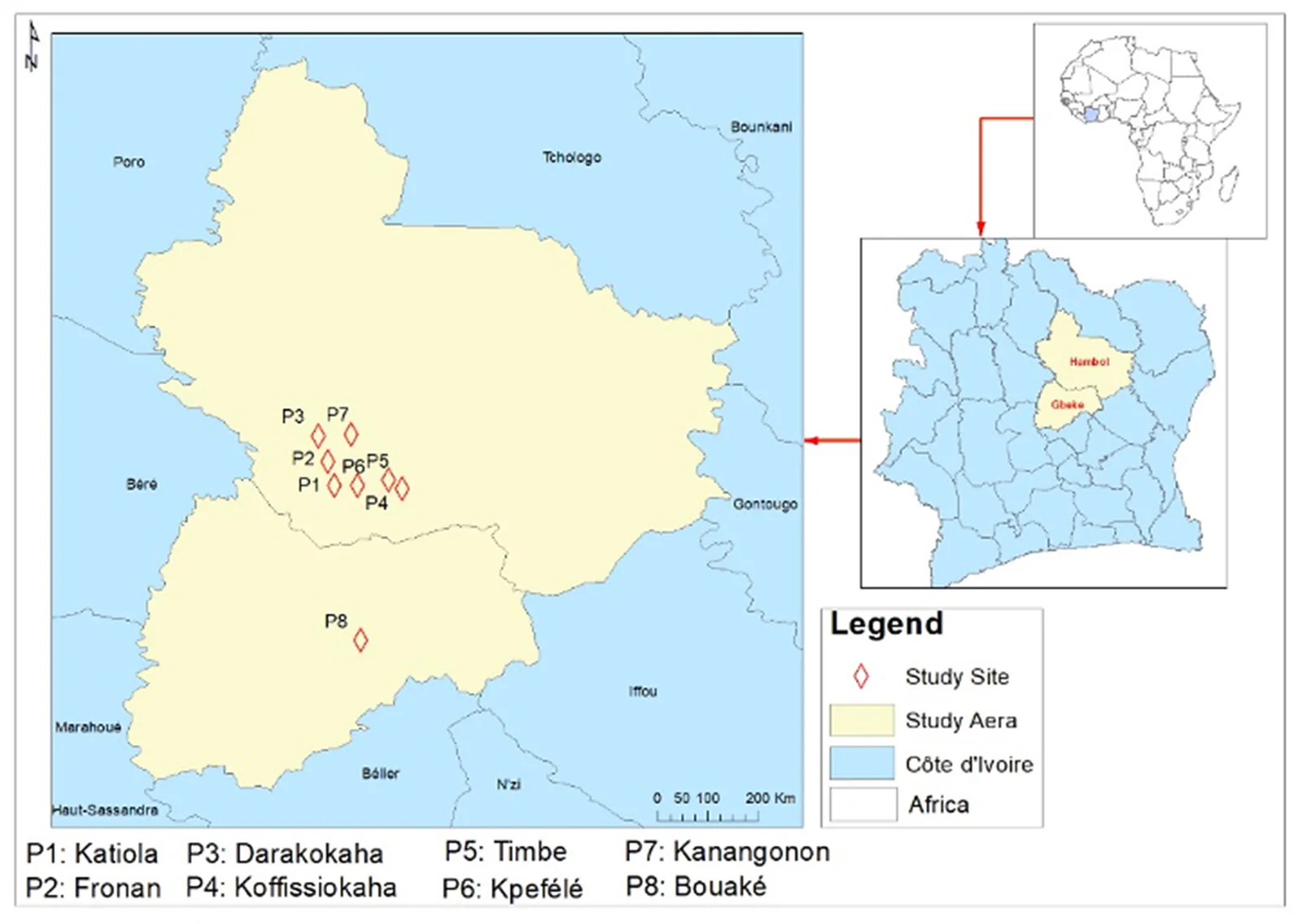
Study sites showing both health districts of Hambol and Gbêkê.

A cross-sectional study was conducted from 19–27 of March 2024 in the Hambol health district, located in north-central Côte d'Ivoire. The objective was to assess the knowledge and practices of animal and human healthcare workers regarding brucellosis and other common febrile illnesses. The results of this initial phase were used to inform the design of a targeted interprofessional education (IPE) intervention aimed at strengthening healthcare workers' capacity to identify and manage human brucellosis case the Hambol and Gbêkê health districts (Figure 1).

A non-probability sampling method was used to recruit participants from both the veterinary and human health sectors. Animal health professionals were selected from the staff of the Regional Directorate of the Ministry of Animal and Fisheries Resources. All animal health workers operating in the Hambol district were invited to participate. Human healthcare workers were recruited from the Maternal and Child Health (MCH) unit of the Regional Hospital Center (CHR) in Katiola, as well as from the Urban Health Centers (CSUs) in Fronan and Timbé and rural Health Centers (CSRs) in Darakokaha, Kanangonon, Koffisiokaha, and kpélé. Participation was voluntary, and all respondents provided written inform consent.

Eligible participants included healthcare professionals actively working in human or animal health services within the study area and willing to provide informed consent. Individuals not directly involved in healthcare delivery or unavailable during the study period were excluded. Participants were approached through their respective institutions and invited to participate voluntarily. No eligible animal health professionals declined participation. Information on the number of invited human healthcare workers who declined participation was not systematically recorded.

Consistent with the study's pilot feasibility and proof-of-concept design, the small sample size and short follow-up period were considered appropriate for generating preliminary evidence. While not intended to support definitive conclusions regarding effectiveness, the sample was sufficient to identify key knowledge gaps among healthcare professionals and to inform the design and implementation of the interprofessional education intervention.

### Data collection tool

Data were collected using a semi-structured questionnaire developed specifically for this study and pre-tested for clarity and contextual relevance. Howver, formal psychometric evaluation, including assessment of content validity and internal consistency reliability, was not conducted. by a multidisciplinary team. The questionnaire gathered information on participants' knowledge and practices regarding brucellosis. It also included sections on participants' professional background, years of experience, and prior exposure to zoonotic disease training. The tool consisted primarily of closed-ended and multiple-choice questions and was organized into the following domains: (1) general knowledge of brucellosis; (2) transmission pathways; (3) clinical symptoms and complications; and (4) diagnostic approaches. The baseline survey included items exploring perceived causes of fever and miscarriage in the region.

The tool was pre-tested to ensure clarity and contextual relevance. Minor revisions were made following pilot testing outside the study area.

### Development of a training module, implementation and impact

Findings from the initial assessment informed the development of context-specific brucellosis training module designed to improving healthcare workers knowledge and awarness in disease recognition, reporting, and intersectoral collaboration. The module was developed through a transdisciplinary collaboration process involving human and animal healthcare workers, epidemiologists, social scientists, and communication experts. The module content was reviewed and validated collaboratively by research team and partners from Tanzania, ensuring consistency with regional One Health training frameworks, The training emphasized teamwork, case-based learning, and mutual understanding of professional roles across disciplines.

The training was structured around clearly defined learning objectives. Participants were expected to: (i) recognize brucellosis as a differential diagnosis in febrile illness and reproductive complications; (ii) identify major transmission pathways; (iii) describe basic diagnostic and referral principles; (iv) understand prevention and control strategies; and (v) strengthen interprofessional communication within a One Health framework. The training emphasized teamwork, case-based learning, and mutual understanding of professional roles across disciplines.

### Training delivering

The IPE training was delivered during a one-day workshop on the 13^th^ of February 2025. Although the intervention was brief, it was designed as a structured and focused capacity-building exercise to assess short-term learning gains and shifts in collaborative attitudes. The workshop included: (i) an introductory plenary session on brucellosis epidemiology and One Health principles; (ii) case-based discussions among professional; (iii) problem-solving exercises based on febrile illness and abortion scenarios; and (iv) facilitated discussions on intersectoral coordination and reporting pathways.

The training was preceded by a co-design phase and included both pre- and post-training assessments using the same questionnaires to measure changes in knowledge. Participants were selected from the Bouaké Nursing School, the Faculty of Medicine of University Alassane Ouattara, the health districts of Gbêkê (Bouaké) and Hambol (Katiola), the Ministry of Animal and Fisheries Resources, and the Center d'Entomologie Médicale et Vétérinaire (CEMV). Prior to implementation, the questionnaire was pre-tested among individuals not involved in the training to allow for appropriate modifications. The training sessions were highly interactive, encouraging participants to share their professional experience, discuss real-world challenges related to zoonotic diseases, and identify opportunities for intersectoral collaboration within a One Health framework.

To assess immediate learning gains, the post-training evaluation was condicted on the same day, approximately two hours after completion of the training. The short interval between the intervention and the post-assessment was intentionally chosen to measure immediate knowledge acquisition and participants' perceptions of the relevance of the training, rather than knowledge retention or longer-term changes in behavior and practice. As such, the evaluation focused on short-term educational outcomes, recognizing that sustained impacts on clinical practice, surveillance activities, and intersectoral collaboration were beyond the scope of this pilot feasibility and proof-of-concept study.

### Data management and analysis

All collected data were cleaned and analyzed using STATA software, version 17. To assess participant knowledge gaps, categorical variables were summarized using frequencies and percentages. Differences between groups were assessed using the Chi-square test or Fisher's exact test, as appropriate.

For the training evaluation, a composite knowledge score was calculated for each participant. Each correct response was assigned one point, while incorrect or missing responses were scored as zero. Domain-specific scores were obtained by summing the number of correct responses within each knowledge domain. The assessment covered five domains: general knowledge of brucellosis (maximum score = 9 points), transmission pathways (14 points), clinical symptoms and complications (6 points), diagnostic approaches (2 points), and prevention and control strategies (5 points). The overall knowledge score was derived by summing the scores across all domains, with no weighting applied, such that higher scores reflected greater knowledge of brucellosis and its management. Domain-specific and overall scores were compared before and immediately after the training to assess short-term learning gains.

The difference between pre- and post-training scores was used to assess the impact of the training. These scores were summarized using mean and standard error. Paired *t-tests* were conducted to determine the statistical significance of observed improvements. For all tests, a *p*-value ≤ 0.05 was considered statistically significant.

All participants completed pre- and post-training and were included in the paired analysis.

## Results

Assessment of animal and human healthcare workers' knowledge gaps and capacity in the diagnosis and management of brucellosis cases.

### Participants profile

Fifty-four participants took part in the study and provided complete data, including 43 human healthcare workers and 11 animal health workers. Among them, there were 32 men and 22 women, with an average age of 39 years (ranging from 26 to 56 years). Their average professional experience was 7 years, including 6 years in the Hambol region.

### Human health

The most frequently encountered human diseases in health centers are malaria (33.3%), followed by typhoid fever (11.1%), meningitis (6.6%), HIV/AIDS (3.7%), tuberculosis (1.9%), and other infections (16.7%) such as respiratory infections, gastroenteritis, and malnutrition.

Regarding causes of febrile illnesses in the region, malaria was the most frequent (98.1%), followed by typhoid fever (41.5%), influenza (32.1%), and respiratory tract infections (30.2%). Other important causes included diarrhea (24.5%), urinary tract infections (22.6%), tuberculosis (15.1%), and gastroenteritis (11.3%). Less frequent causes included otitis media (3.8%) and dengue (1.9%). Notably, brucellosis was not mentioned.

Abortions or miscarriages in the first trimester of pregnancy in women were a concern among respondents. Out of the 54 participants, 31 (57.4%) reported being aware of abortions or miscarriages occurring in the first trimester of pregnancy in the region. In contrast, 11 participants (20.4%) stated they were not aware of such events, while 12 participants (22.2%) did not know or chose not to answer the question.

According to those who reported such cases, malaria and lack of prenatal follow-up were identified as major and recurrent causes of abortion or miscarriage during the first 3 months of pregnancy. Repeated episodes of fever, urinary and genital tract infections also emerged as major causes, alongside other factors such as malnutrition, toxoplasmosis, and substance use (alcohol and drugs). Brucellosis was not mentioned as a potential cause of miscarriage by respondents.

### Animal health

In the Hambol Region, livestock farming is a widespread activity. Cattle and pigs are the most represented species, followed by sheep and goats. The data collected on the most reported animal diseases in the study area revealed important epidemiological trends. Foot-and-mouth disease (FMD) and contagious bovine pleuropneumonia (CBPP) were the most frequently reported conditions, each accounting for 14.8% of cases. In contrast, diseases such as “peste des petits ruminants” (PPR) and lumpy skin disease (LSD) were reported less frequently (each at 3.7%). Brucellosis and tick- borne diseases, each reported at 1.9%, appeared underrepresented. Finally, the “Other” diseases category, fasciolosis, swine fever, and bovine tuberculosis represented 5.6% of reported cases.

Among the 54 respondents, only five individuals (veterinarians) (9.3%), reported abortions in livestock. A slightly smaller number, four respondents (7.4%), stated that they had not observed any abortion events. Strikingly, the vast majority, 45 respondents (83.3%), answered “don't know”. However, all veterinarians reported observing clinical signs in livestock suggestive of brucellosis, including orchitis and decreased fertility. While such symptoms were not pathognomonic and may be associated with a range of infectious or non-infectious conditions, none of the cases were laboratory-confirmed, as no diagnostic testing was conducted to identify the underlying etiology.

### Knowledge of zoonoses and brucellosis

Data analysis highlighted the participants' knowledge of zoonoses, i.e., diseases transmissible from animals to humans. Among the 49 individuals (90.7%) who had knowledge of zoonoses, 61.2% were men and 38.8% were women. Although men appeared to be more represented, this difference was not statistically significant (*p* = 0.36). In terms of age, individuals aged 25 to 35 years were the most represented (36.7%), followed by those aged 35 to 45 (34.7%), and those aged 45 and above (28.6%), without significant impact on zoonosis knowledge across age groups (*p* = 0.91). The participants' professions somehow influenced their understanding of zoonoses. Human healthcare workers appeared more involved with or exposed to zoonotic disease, with 77.6% recognizing them, compared to only 22.5% of veterinarians. However, this difference was not statistically significant (*p* = 0.24).

Among respondents who acknowledged zoonoses, only 38.8% (35.8% of all respondents) mentioned brucellosis as a zoonotic disease. The findings showed a substantial gap in zoonotic disease awareness between human and animal health sectors. Only 23.3% of human healthcare workers reported knowledge of brucellosis, while 81.3% of veterinarians were familiar with the disease. Statistical analysis confirmed this disparity, with a highly significant *p*-value (< 0.001). The findings also highlighted not only the important role of professional but also experience in understanding brucellosis. Most participants had substantial professional experience, with 47.4% having over 10 years and 31.6% between 5 and 9 years in the field. A similar pattern was observed in the Hambol region, where 31.6% had over 10 years of local experience and another 31.6% had 5 to 9 years. Only 5.3% were beginners in either category. Statistical analysis showed a significant association between experience and knowledge of brucellosis, with those having more than 10 years of overall experience being more knowledgeable (47.4%; *p* = 0.02), and a similar trend observed for local experience in the Hambol region (31.6%; *p* = 0.04).

Strengthening human healthcare workers' knowledge and capacity to manage human brucellosis.

### Characteristics of training participants

Table 1 summarizes the sociodemographic and institutional profile of the 22 participants involved in training. Most participants were male (17 individuals, 77.3%), while only 5 were female (22.7%). Most participants were human healthcare workers (77.3%), while veterinary workers represented 22.7% of the sample. Participants came from five different institutions: CEMV (Center d'Entomologie Médicale et Vétérinaire), (45.5%); health districts of Hambol (22.7%) and Gbêkê (18.2%), LANADA (National Laboratory for Agricultural Development Support), (9.1%). One participant (4.6%) came from the faculty of medical science.

**Table 1:** Distribution of participants by gender, occupation, and institution.

Variables	*n* (%)
**Gender**	
Males	17 (77.3)
Females	5 (22.7)
**Occupation**	
Human healthcare worker	17 (77.3)
Veterinary worker	5 (22.7)
**Institution**	
Health district of Gbêkê	4 (18.2)
Health district of Hambol	5 (22.7)
LANADA	2 (9.1)
Medical science training unit	1 (4.6)
CEMV	10 (45.5)
**TOTAL**	**22 (100)**

### Impact of the training on brucellosis knowledge

Table 2 summarizes the impact of the immediate post-intervention gains within a short time frame. The 22 participants were assessed both before and after the training, with mean scores recorded across several knowledge domains, including overall knowledge, basic understanding of brucellosis, transmission, symptoms, diagnosis, and control strategies. The results showed a significant improvement in the overall general knowledge score, which increased from 34.5 (95% IC = 33.9–35.1) to 37.0 (95% IC = 36.4–40.1) (*p* < 0.005). This improvement was statistically significant among human health workers (*p* < 0.005), but not among veterinary workers (*p* = 0.09), Knowledge of brucellosis symptoms significantly improved, increasing from 4.9 (95% IC = 4.7–5.3) to 5.9 (95% IC = 5.8–6.0), (*p* < 0.005), especially among human healthcare workers (*p* < 0.005). Knowledge of transmission, diagnosis, and control strategies did not show significant changes after training (*p* > 0.2). The score for basic disease knowledge was already high before the training (6.9/9) and remained stable afterward (7.0), with no significant difference (*p* = 0.69).

**Table 2:** Effect of training on brucellosis knowledge among human and animal healthcare workers: comparison of pre- and post-training scores across knowledge domains.

Variables	Before the training		After the training		*t* (ddl)	*p*-value
	Mean (95% IC)	Min-max	Mean (95% IC)	Min-max		
	**General knowledge on brucellosis**					
Overall score	34.5 (33.9–35.1)	28–40	37.0 (36.4–40.1)	34–41	4.1 (21)	**< 0.005**
**Occupation**						
Human healthcare worker	34.8 (34.2–35.4)	29–39	37 (36.5–37.5)	34–41	3.4 (16)	**< 0.005**
Veterinary worker	33.8 (31.7–35.9)	28–40	36.8 (35.8–37.8)	34–40	2.2 (4)	0.09
**Knowledge of brucellosis**						
Overall score	6.9 (6.7–7.1)	4–9	7.0 (6.9–7.1)	7–7	0.4 (21)	0.69
**Occupation**						
Human healthcare worker	6.9 (6.7–7.1)	4–8	7 (6.9–7.1)	7–7	0.5 (16)	0.7
Veterinary worker	7.0 (6.4–7.6)	5–9	7 (6.9–7.1)	7–7	0.0 (4)	1.0
**Transmission**						
Overall score	12.0 (11.7–12.3)	8–14	12.4 (12.2–12.6)	10–14	1.3 (21)	0.2
**Occupation**						
Human healthcare worker	12.0 (11.6–12.4)	8–14	12.3 (12.0–12.6)	10–14	0.8 (17)	0.44
Veterinary worker	11.8 (11.0–12.6)	9–13	12.6 (12.1–13.1)	11–14	1.4 (4)	0.24
**Symptoms**						
Overall score	4.9 (4.7–5.3)	3–6	5.9 (5.8–6.0)	4–6	4.1 (21)	**< 0.005**
**Occupation**						
Human care worker	5.0 (4.7–5.3)	3–6	6 (5.9–6.1)	6–6	3.7 (16)	**< 0.005**
Veterinary worker	4.6 (4.0–5.2)	3–6	5.6 (5.2–6.0)	4–6	1.6 (4)	0.19
**Diagnostic**						
Overall score	0.8 (0.7–0.9)	0–2	0.6 (0.4–0.8)	0–2	−0.8 (21)	0.41
**Occupation**						
Human healthcare worker	0.9 (0.7–1.1)	0–2	0.7 (0.5–0.9)	0–2	−0.8 (16)	0.41
Veterinary worker	0.4 (0.0–0.8)	0–2	0.4 (0.0–0.8)	0–2	NA	NA
**Control strategy**						
Overall score	3.8 (3.7–3.9)	3–5	4.0 (3.8–4.2)	3–5	0.8 (21)	0.42
**Occupation**						
Human healthcare worker	3.8 (3.6–4.0)	3–5	3.9 (3.7–4.1)	3–5	0.3 (16)	0.75
Veterinary worker	3.8 (3.6–3.4)	3–4	4.2 (3.8–4.6)	3–5	1.0 (4)	0.37

Bold values mean statistically significant

**Figure 2:**
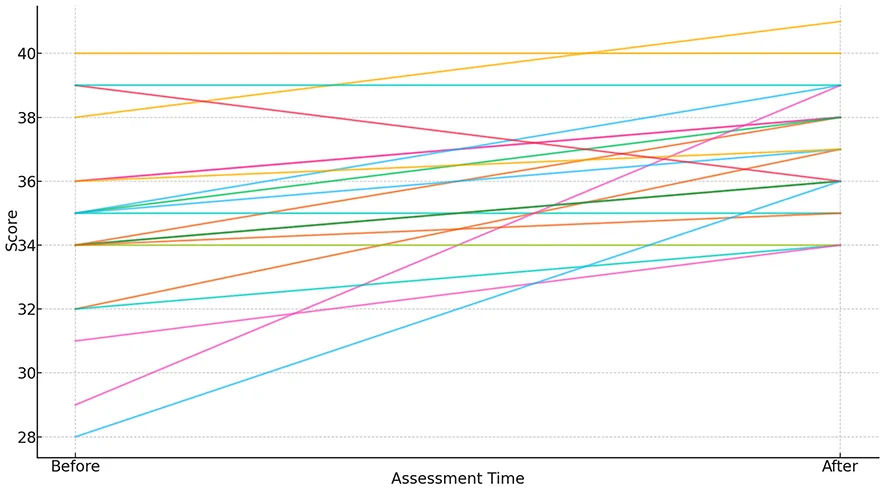
Individual changes in general knowledge scores before and after training.

Figure 2 illustrates the change in general knowledge scores for each of the 22 participants. Most participants demonstrated an increase in their scores following the training, indicating an overall improvement in knowledge. A few participants started with lower baseline scores and showed substantial gains, while others who already had high baseline knowledge exhibited minimal change, possibly reflecting a ceiling effect. Only one participant showed a slight decrease.

**Figure 3:**
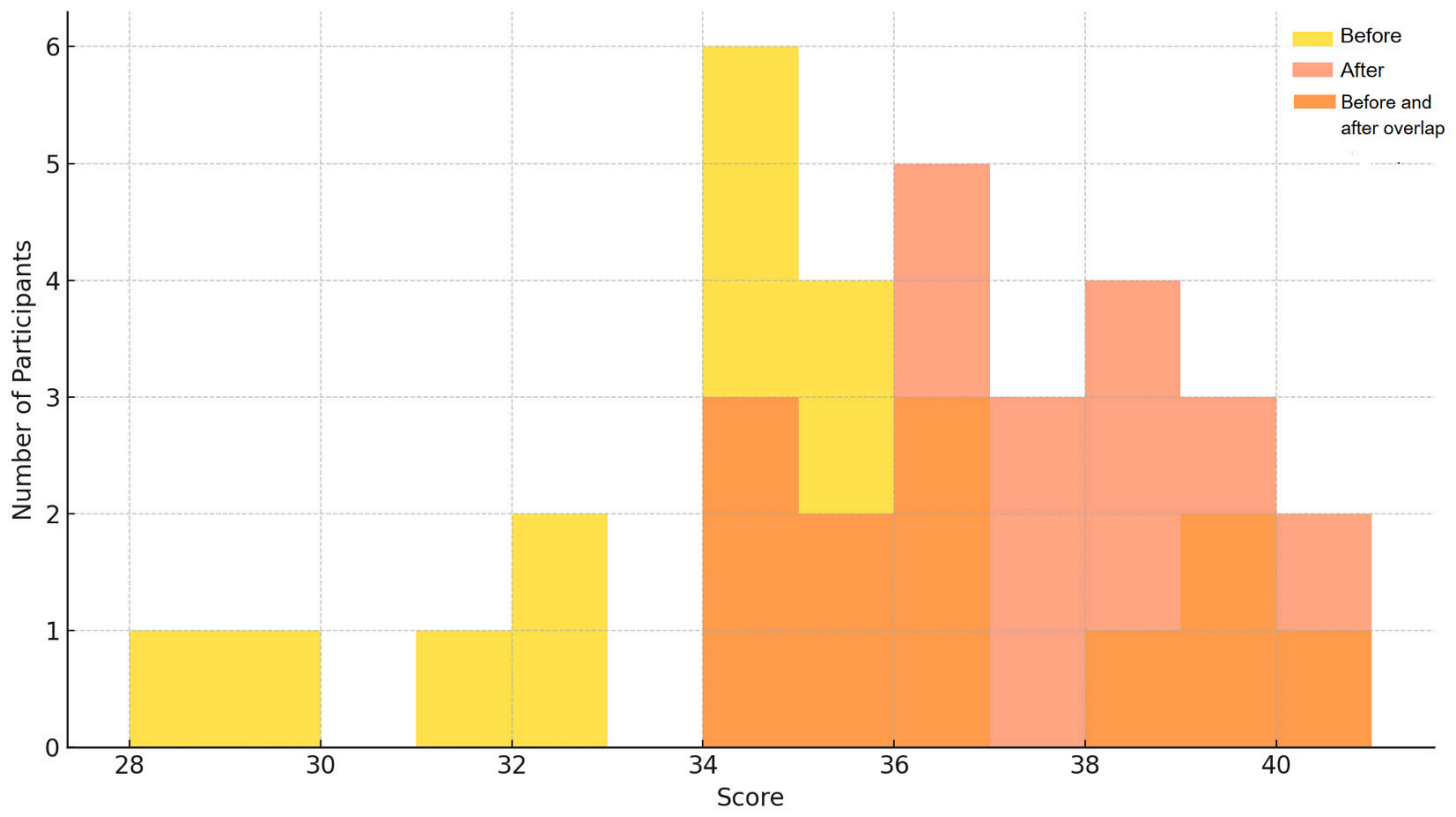
Distribution of general knowledge scores before and after the training session.

The Figure 3 displays the distribution of general knowledge scores on brucellosis among the 22 participants, assessed before (yellow) and after (orange) the training. Before the training, scores were more widely dispersed, with several participants scoring below 34. In contrast, post-training scores shifted markedly to the right, clustering between 36 and 41. This shift reflects an overall improvement in knowledge, with a clear reduction in lower scores and an increase in the number of participants achieving higher scores.

## Discussion

This pilot study explored the knowledge and practices of human and animal healthcare workers regarding brucellosis and assessed the short-term impact of an interprofessional education (IPE) module designed to improve disease recognition and management. As a feasibility and proof-of-concept initiative, the study aimed primarily to knowledge gaps and evaluate immediate learning outcomes rather than to demonstrate long-term effectiveness.

The study contributed to the limited evidence IPE for zoonotic disease control in sub-Saharan Africa. To our knowledge, it is among the first pilot feasibility and proof-of-concept studies in Côte d'Ivoire to apply an IPE approach within a One Health framework to address knowledge gaps related to brucellosis among human and animal health professionals. Unlike conventional training initiatives, the intervention was informed by a baseline assessment of local knowledge needs and was co-designed by stakeholders from multiple disciplines. The study provided preliminary evidence on the feasibility of implementing a One Health-oriented IPE intervention in a resource-limited setting and highlights important diagnostic blind spots that may contribute to the underrecognition of brucellosis. These findings offer a foundation for the development and evaluation of larger, longitudinal IPE programs aimed at strengthening collaboration between human and animal health sectors in the management of neglected zoonotic diseases.

Consistent with evidence from other sub-Saharan African countries, the study revealed significant deficiencies in brucellosis knowledge among human healthcare workers, particularly regarding transmission pathways, symptom recognition, and its association with spontaneous abortions (3, 12, 13). Only a small proportion of participants associated brucellosis with febrile illness or reproductive complications, while most cited malaria and typhoid as the main causes of fever. These finding highlight importance blind spots but should be interpreted in light of the limited, non-random sample drawn from a single region. Nevertheless, this under recognition aligns with previous findings that brucellosis remains underdiagnosed in febrile patients due to its nonspecific clinical presentation and lack of diagnostic tools in endemic regions (14, 15). In contrast, animal health professionals demonstrated a higher level of awareness, likely due to their more frequent exposure to livestock cases and disease management training (16, 17).

The lack of routine diagnostic testing for *Brucella* species in local health facilities further exacerbates this diagnostic gap and perpetuates underreporting (18). None of the human healthcare workers interviewed mentioned brucellosis as a possible cause of recurrent abortions, even though such associations are well documented in endemic settings) (19). This finding underscores the importance of integrating zoonotic disease awareness into reproductive health services and improving diagnostic capacity at the primary healthcare level.

The interprofessional training module developed in this study demonstrated measurable improvement in participants' overall knowledge, especially in symptom recognition and awareness of zoonotic risks. These results are consistent with prior studies showing that short, targeted educational interventions can significantly improve health professionals' competencies in zoonosis prevention and management (20–22). Moreover, the interprofessional format of the training fostered collaboration between medical, veterinary, and public health professionals; an essential element of One *Health* practice that enhances coordination in surveillance and response (23, 24). However, the improvements observed represent immediate post-training and not necessarily imply sustained changes in clinical behavior, diagnostic practice, or surveillance performance.

Although the overall knowledge score increased significantly following the training, the magnitude of improvement was modest, rising from 34.5 to 37.0 points. While a 2.5-point increase may appear small, it is noteworthy given the short duration of the interprofessional education (IPE) intervention and suggests measurable gains in participants' understanding of brucellosis and One Health concepts. The relatively modest improvement may reflect the diverse professional backgrounds of participants and the variability in baseline knowledge across sectors. In addition, some domains had relatively high pre-training scores, leaving limited room for improvement and suggesting a possible ceiling effect, particularly among veterinary professionals who were generally more familiar with brucellosis. Despite the small absolute increase, the findings indicate that the IPE intervention contributed to reinforcing existing knowledge and addressing specific knowledge gaps across professional groups.

Nevertheless, the absence of a control group limits the ability to attribute these improvements solely to the intervention. Alternative explanations, including testing effects, recall bias, social desirability bias, and the Hawthorne effect, may have influenced participants' responses. Therefore, the observed changes should be interpreted as being associated with participation in the training rather than as definitive evidence of causality. Future studies should incorporate comparison groups and assess longer-term knowledge retention, as well as the impact of training on intersectoral collaboration, disease surveillance, and One Health practice.

The limited improvement in diagnostic and control knowledge suggests that a single-day workshop is insufficient for developing deeper competencies require for effective clinical and public health practice. This highlights the need for iterative, longitudinal, and practice-oriented training models to achieve meaningful and sustained system-level impact. A modular training framework; complemented by mentoring, simulation-based learning, and supervised field practice; would likely support stronger knowledge consolidation and more durable behavioral change (25, 26).

As a pilot initiative, this study's small, non-random sample, absence of control group, and short follow-up period limit the generalizability of findings. Furthermore, the evaluation focused exclusively on knowledge outcomes and did not assess behavioral change, clinical decision-making, patient outcomes, or intersectoral reporting performance. Nevertheless, it fulfilled its primary goal: to test the feasibility and short-term effectiveness of IPE for zoonotic disease management in Côte d'Ivoire. The lessons learned provide a foundation for scaling up the intervention across additional districts, with a broader participant base and longitudinal evaluation to assess knowledge retention and behavioral change.

Overall, the findings reinforce the need to embed interprofessional and One Health training approaches into national health systems, while recognizing that sustainable impact requires institutional support, repeated training cycles, and integration into continuing professional education frameworks. Future research should incorporate qualitative methods, including in-depth interviews, to better understand barriers to diagnostic recognition and interprofessional collaboration. Such approaches would help capture the contextual factors shaping clinical decision-making, perceptions of zoonotic disease risks, and the operational challenges faced by professionals in both the human and animal health sectors. They would also provide valuable insight into communication pathways, coordination processes, and structural constraints that may hinder the effective implementation of One Health practices at the local level.

This study has several limitations that should be considered when interpreting the findings. First, as a pilot feasibility and proof-of-concept study, it involved a relatively small, non-random sample drawn from a limited number of institutions and a single geographic region, which may restrict the generalizability of the results. Second, the absence of a control group prevents attribution of the observed knowledge gains exclusively to the intervention. Improvements may have been influenced by repeated exposure to the questionnaire (testing effect), recall bias, social desirability bias, or Hawthorne effects associated with participation in the training. Third, knowledge was assessed immediately after the intervention, approximately 2 h following training completion. Consequently, the study measured short-term knowledge acquisition rather than knowledge retention, behavioral change, clinical practice modification, or improvements in surveillance performance. Fourth, the evaluation focused exclusively on knowledge outcomes and did not assess diagnostic accuracy, referral practices, intersectoral collaboration, patient outcomes, or broader health system impacts. Fifth, although the questionnaire was developed through a multidisciplinary co-design process and pre-tested for clarity and contextual relevance, formal assessments of content validity and reliability (e.g., internal consistency measures such as Cronbach's alpha) were not conducted. Finally, baseline scores were already high in some knowledge domains, particularly general knowledge of brucellosis, suggesting a potential ceiling effect that may have limited the magnitude of measurable improvement following the intervention. Despite these limitations, the study achieved its primary objective of identifying knowledge gaps and generating preliminary evidence on the feasibility of implementing interprofessional education within a One Health framework in Côte d'Ivoire.

## Conclusion

This pilot study revealed critical knowledge gaps about brucellosis among human healthcare workers in the Hambol region of Côte d'Ivoire and demonstrated the feasibility of implementing IPE and provided preliminary evidence of immediate knowledge improvement. While animal health professionals displayed stronger baseline understanding, human healthcare workers rarely considered brucellosis in cases of fever or miscarriage, highlighting an important diagnostic blind spot in clinical practice.

The 1-day IPE training significantly improved general understanding and symptom recognition, showing that brief, structured interventions can promote awareness and cross-sectoral collaboration. However, these improvements were measured immediately after the intervention, and the study did not assess long-term knowledge retention, behavioral change, or clinical practice modification. Therefore, the finding should be interpreted as preliminary evidence rather than definitive proof of effectiveness and may serve to design larger and robust studies.

Future efforts should focus on integrating brucellosis and other zoonoses into continuous professional education, expanding diagnostic capacity at primary healthcare and veterinary levels, and strengthening cross-sectoral communication systems. Longitudinal evaluation and larger, multi-site studies will be essential to determine whether IPE-based approaches translate into sustained improvements in surveillance, diagnosis, and patient outcomes. Though limited in scope, this study provides a valuable model for scaling up One Health educational initiatives to improve zoonotic disease preparedness in Côte d'Ivoire and similar settings.

## Data Availability

The raw data supporting the conclusions of this article will be made available by the authors, without undue reservation.
