# Supplementary material for: Breaking professional silos to combat brucellosis: a pilot feasibility and proof-of-concept study of interprofessional education to strengthen healthcare capacity in Côte d'Ivoire
Source: Front Vet Sci. 2026 Jul 30;13:1742755. doi: 10.3389/fvets.2026.1742755 (PMC13501971; doi:10.3389/fvets.2026.1742755)
Supplement: Supplementary file 1 [file DataSheet_1.pdf]

Questionnaire

Improving the Capacity of Health Professionals to Diagnose and Manage Human Brucellosis (RAPIDNESS)

Questionnaire No: ____

Investigator's name: ________________________

Service ID: ____

Locality: ____________________

Type of service: ____

1: Rural health center (RHC)
2: Urban health center (UHC)
3: General hospital (GH)
4. Veterinary service (VS)

GPS position: _____________________

Interview start time: H:____ mm______

I. Respondent Information

Full name: ________________________

Gender: M /__; F /__

Age (years): ______

Profession:

- a. Physician
- b. Nurse
- c. Midwife
- d. Laboratory technician
- e. Community health worker (CHW),
- f. Veterinary

Years of experience: ____

Years of experience in the locality (Hambol region): ____

II. General Knowledge on Brucellosis

1. Do you know of diseases transmissible from animals to humans? Yes/__; No/__

2. Do you know about brucellosis? Yes/__; No/__

3. Have you ever heard of brucellosis? Yes/__; No/__; if No, go to question 15.

4. If yes, under what circumstance did you hear about brucellosis?

    a. At school during training Yes/__; No/__

    b. During a workshop/seminar Yes/__; No/__

    c. During professional practice Yes/__; No/__

5. Have you ever encountered a case of brucellosis? Yes/__; No/__

6. If yes, what type of case? Human /__; Animal /__; Both /__

7. Do you think brucellosis is a:

    a. Serious disease Yes/__; No/__

    b. Quite serious Yes/__; No/__

    c. Very serious Yes/__; No/__

    d. Not serious Yes/__; No/__

    e. Don't know Yes/__; No/__

8. What type of disease is brucellosis?

    a. Infectious Yes/__; No/__

    b. Contagious Yes/__; No/__

    c. Transmissible Yes/__; No/__

    d. Metabolic Yes/__; No/__

    e. Don't know Yes/__; No/__

9. Do you know the pathogen of brucellosis?

    a. Virus Yes/__; No/__

    b. Bacterium Yes/__; No/__

    c. Parasite Yes/__; No/__

d. Fungus Yes/__; No/__

e. Don't know Yes/__; No/__

10. Do you know the mode of transmission of brucellosis?

a. Animal to human Yes/__; No/__

b. Close contact with livestock Yes/__; No/__

c. Consumption of unpasteurized dairy products Yes/__; No/__

d. Other, specify: __________

e. Don't know Yes/__; No/__

11. Signs and symptoms of brucellosis in humans:

a. Fever Yes/__; No/__

b. Headache Yes/__; No/__

c. Weight loss Yes/__; No/__

d. Loss of appetite Yes/__; No/__

e. Joint and bone pain Yes/__; No/__

f. Back pain Yes/__; No/__

g. Diarrhea Yes/__; No/__

h. Cough i. Anemia Yes/__; No/__

j. Miscarriage or stillbirth Yes/__; No/__

k. Male infertility Yes/__; No/__

l. Others, specify: _____ m.

Don't know Yes/__; No/__

12. Signs and symptoms of brucellosis in animals:

a. Abortion or stillbirth Yes/__; No/__

b. Birth of weak offspring Yes/__; No/__

c. Anemia (paleness) Yes/__; No/__

d. Weight loss Yes/__; No/__

e. Loss of appetite Yes/__; No/__

f. Male infertility Yes/__; No/__

g. Retained placenta Yes/__; No/__

h. Hygroma Yes/__; No/__

i. Orchitis Yes/__; No/__

j. Behavioral disorders Yes/__; No/__

k. Reduced milk production Yes/__; No/__

l. Lameness Yes/__; No/__

m. Other

n. Don't know Yes/__; No/__

13. Do you recommend the brucellosis test to your patients/farmers? Yes/__; No/__

14. If No, why? __________________________

15. Do you know the diagnostic tests for brucellosis?

a. Rose Bengal agglutination test Yes/__; No/__

b. Rapid diagnostic test Yes/__; No/__

c. Molecular test (PCR) Yes/__; No/__

d. ELISA Yes/__; No/__

e. Don't know Yes/__; No/__

16. Which of these tests do you practice?

a. Rose Bengal test Yes/__; No/__

b. Rapid test Yes/__; No/__

c. PCR Yes/__; No/__

d. ELISA Yes/__; No/__

e. None Yes/__; No/__

17. Would you like to receive training on brucellosis? Yes/__; No/__

18. Would you like to share your knowledge of brucellosis with other human and animal health professionals? Yes/__; No/__

19. What are the most common human diseases in your locality? a. Malaria b. Typhoid fever c. Tuberculosis d. HIV/AIDS e. Meningitis f. Others, specify: _________

20. What are the most frequent clinical signs in humans?

a. Fever b. Diarrhea Yes/__; No/__

c. Cough d. Miscarriage Yes/__; No/__

e. Stillbirth Yes/__; No/__

f. Joint pain Yes/__; No/__

g. Fatigue Yes/__; No/__

h. Headache Yes/__; No/__

i. Malnutrition Yes/__; No/__

j. Anemia Yes/__; No/__

21. What are the most frequent livestock diseases in your locality?

a. Foot-and-mouth disease Yes/__; No/__

b. Brucellosis Yes/__; No/__

c. Contagious bovine pleuropneumonia Yes/__; No/__

d. Tick-borne diseases Yes/__; No/__

e. Rinderpest Yes/__; No/__

f. Peste des petits ruminants Yes/__; No/__

g. Lumpy skin disease Yes/__; No/__

h. Others, specify: _________

22. What are the most frequent clinical signs in animals?

a. Abortions Yes/__; No/__

b. Skin nodules Yes/__; No/__

c. Weight loss Yes/__; No/__

d. Infertility Yes/__; No/__

e. Hygromas Yes/__; No/__

f. Orchitis Yes/__; No/__

g. Fever Yes/__; No/__

h. Hair loss Yes/__; No/__

i. Reduced milk production Yes/__; No/__

j. Loss of appetite Yes/__; No/__

Interview end time: H:_____ mm_______
