# Supplementary material for: Breaking professional silos to combat brucellosis: a pilot feasibility and proof-of-concept study of interprofessional education to strengthen healthcare capacity in Côte d'Ivoire
Source: Front Vet Sci. 2026 Jul 30;13:1742755. doi: 10.3389/fvets.2026.1742755 (PMC13501971; doi:10.3389/fvets.2026.1742755)
Supplement: Supplementary file 2 [file DataSheet_2.pdf]

Questionnaire – Ante and post training

Improving the Capacity of Health Professionals to Diagnose and Manage Human Brucellosis (RAPIDNESS)

Brucellosis Knowledge Assessment Test

Section 1: General Participant Information

1. ID (Identification Code): ____

2. Gender

Select only one answer.

- Male
- Female

3. Profession

Select only one answer

- Physician
- Nurse
- Midwife
- Nursing Assistant
- Laboratory Technician
- In Training/Student
- Veterinary Agent
- Other: ______

4. Affiliation/Organization

Select only one answer.

- Hambol Health District
- Gbéké Health District
- INFAS (Nursing school)
- Medical Sciences
- LANADA (National Agriculture Laboratory)
- Other: _____

5. Phone Contact: ______

6. Knowledge Assessment

Select only one answer.

- Before training
- After training

Section 2: General Knowledge about Brucellosis

7. Have you ever heard of brucellosis? Yes__/No___

8. If yes, where did you hear about it?
Select all that apply
- Training Yes__/No___
- Media
- Colleagues
- Veterinarian training
- Other: ______

9. Is brucellosis a contagious disease? Yes__/No___/ I don’t know

10. What type of agent causes brucellosis?
Select only one answer.
- Bacteria
- Virus
- Parasites
- Fungi
- I don't know

11. Which of these pathogens is responsible for brucellosis?
Select only one answer.
- *Brucella* species
- *Salmonella typhi*
- *Escherichia coli*
- *Mycobacterium tuberculosis*
- I don't know
- Other: _____

12. Does brucellosis affect both humans and animals? Yes__/No___/ I don’t know

13. If yes, which animals can be affected by brucellosis?
Select all that apply.
- Cattle
- Sheep
- Goats
- Pigs
- Dogs
- None
- I don't know
- Other: _____

14. Which Brucella species is commonly found in cattle?
Select only one answer.
- *Brucella abortus*
- *Brucella melitensis*

- *Brucella suis*
- *Brucella canis*
- I don't know

15. Which Brucella species is the most pathogenic for humans?
    Select only one answer.
    - *Brucella melitensis*
    - *Brucella abortus*
    - *Brucella suis*
    - *Brucella canis*
    - I don't know

16. Which animal species is primarily affected by Brucella suis?
    Select only one answer.
    - Pigs
    - Sheep
    - Cattle
    - Dogs
    - I don't know

17. Which Brucella species has the highest zoonotic potential?
    Select only one answer.
    - *Brucella melitensis*
    - *Brucella abortus*
    - *Brucella suis*
    - *Brucella canis*
    - I don't know

18. Which region is most often associated with brucellosis outbreaks?
    Select only one answer.
    - Mediterranean Basin
    - North America
    - Arctic Circle
    - Western Europe
    - I don't know

19. Which professions are most frequently associated with brucellosis?
    Select all that apply.
    - Agriculture (Livestock farming)
    - Teaching
    - Veterinary Agent
    - Banking
    - Healthcare Worker
    - Construction Work
    - Slaughterhouse Workers
    - I don't know

- Other: ______

20. What is the economic impact of brucellosis in endemic regions mainly attributed to?
    Select only one answer.
    - Loss of livestock productivity
    - Increased healthcare costs
    - Trade restrictions
    - All of the above

Section 3: Transmission of Brucellosis

21. What are the routes of transmission of brucellosis to humans?
    Select all that apply.
    - Consumption of contaminated dairy products
    - Direct contact between people
    - Airborne transmission
    - Bite from an infected animal
    - I don't know
    - Other: ______

22. Which group is at highest risk of contracting brucellosis?
    Select only one answer.
    - Veterinarians and Livestock Farmers
    - Urban Office Workers
    - Teachers
    - Athletes
    - I don't know

23. What is the main route of transmission of Brucella to humans?
    Select only one answer.
    - Ingestion of contaminated dairy products
    - Person-to-person contact
    - Bites from infected animals
    - Mosquito bites
    - I don't know

24. Which bodily fluid from an infected animal contains the highest concentration of Brucella bacteria?
    Select only one answer.
    - Milk
    - Blood
    - Saliva
    - Sweat
    - I don't know

25. Which type of dairy product is most likely to transmit brucellosis?

Select only one answer.

- Raw milk
- Pasteurized milk
- Flavored yogurt
- Processed cheese
- I don't know

Section 4: Symptoms and Complications of Brucellosis

26. Which clinical symptom is commonly associated with brucellosis in humans?
Select only one answer.

- Intermittent fever
- Skin rash
- Chronic cough
- Severe diarrhea
- I don't know

27. What is the main clinical manifestation of brucellosis in animals?
Select only one answer.

- Abortions in pregnant females
- Severe diarrhea
- Neurological symptoms
- Weight gain
- I don't know

28. Which medical complication of brucellosis requires immediate attention?
Select only one answer.

- Endocarditis
- Mild headache
- Skin rash
- Low blood pressure
- I don't know

Section 5: Diagnosis of Brucellosis**

29. Which diagnostic method is considered the gold standard for brucellosis?
Select only one answer.

- Blood culture
- PCR test
- Rapid diagnostic tests
- ELISA
- I don't know

Section 6: Prevention and Control of Brucellosis

30. Which prevention strategy is crucial for controlling brucellosis in livestock?

Select only one answer.

- Animal vaccination
- Frequent trading of animals
- Avoiding contact with water sources
- Isolation of healthy animals
- I don't know

31. 25) What is the most effective strategy to prevent brucellosis in humans?
    Select all that apply.

- Vaccinate livestock
- Use insect repellent
- Avoid consumption of raw milk
- Wear gloves for gardening
- Avoid raw vegetables
- I don't know

32. What is a key public health measure to reduce the risk of brucellosis?
    Select only one answer.

- Promote pasteurization of dairy products
- Use of mosquito nets
- Increase blood donation
- Encourage a vegetarian diet
- I don't know

33. Which professional groups should be prioritized for brucellosis awareness campaigns?
    Select all that apply.

- Veterinarians and slaughterhouse workers
- Teachers and students
- Retail workers
- Software engineers
- I don't know

Thank you for your participation
